# Cross-Cultural Differences in Caregiving: Investigating the Role of Familism and Social Support

**DOI:** 10.1093/geroni/igab046.1249

**Published:** 2021-12-17

**Authors:** Francesca Falzarano, Jerad Moxley, Karl Pillemer, Sara Czaja

**Affiliations:** 1 Weill Cornell Medicine, Douglaston, New York, United States; 2 Weill Cornell Medicine, New York, New York, United States; 3 Cornell University, Ithaca, New York, United States; 4 Weill Cornell Medicine/Center on Aging and Behavioral Research, New York, New York, United States

## Abstract

Cultural diversity in the United States (US) reflects a demographic shift, with a growing population of minority older adults and a subsequent increase in minority family caregivers providing care to aging adults. Research has demonstrated heterogeneity in the caregiving experience, with increasing focus placed on examining the impact of cultural values on caregiver (CG) outcomes. Familism has been investigated as a driving mechanism of cross-cultural differences in caregiving outcomes, yet prior work examining this relationship has yielded mixed findings. Using the sociocultural stress and coping model as a guiding framework, we examined, in a sample of 243 CGs who participated in the Caring for the Caregiver Network Study, a randomized controlled trial examining a culturally-tailored technology-based psychosocial intervention, the influence of familism and social support on positive aspects of caregiving, depressive symptoms, and caregiver burden. We also examined how these relationships vary as a function of race/ethnicity, the CG’s relationship to the care-recipient, other sociodemographic characteristics (e.g., SES status), and acculturation. Results showed that African American and Hispanic CGs exhibited higher levels of familism compared to Whites. In African Americans, familism predicted higher positive caregiving appraisals, and social support significantly predicted lower burden and depression. In Hispanics, levels of familism varied as a function of acculturation, with lower levels of familism identified among US Hispanic natives. Our findings highlight that cultural beliefs, such as familism, as well as social support may be adaptive in protecting against adverse CG outcomes and point to directions for future culturally congruent, family-centered intervention approaches.

